# P-2086. The Clinical Profile of Patients with Indeterminate Xpert Rifampin Resistance Results in a Single Tertiary Level Institution

**DOI:** 10.1093/ofid/ofae631.2242

**Published:** 2025-01-29

**Authors:** Bettina Gabrielle V Tenorio, Danielle Josefa F Uayan, Kriselle Felicia Lumunsad, Andrea S Estuart, Jose Carlos Ruben R Javier, Cybele Lara R Abad

**Affiliations:** Ateneo School of Medicine and Public Health; NYC Health + Hospitals / Elmhurst, Icahn School of Medicine at Mount Sinai, Woodside, New York; Ateneo School of Medicine and Public Health, Bacoor City, Cavite, Philippines; The Medical City, Paranaque, National Capital Region, Philippines; The Medical City, Paranaque, National Capital Region, Philippines; The Medical City, Paranaque, National Capital Region, Philippines; The Medical City; UP-PGH, Manila, National Capital Region, Philippines

## Abstract

**Background:**

Tuberculosis (TB) is one of the leading causes of morbidity and mortality globally. Since the development of point-of-care tests such as the Xpert MTB/RIF assay, TB diagnosis has improved dramatically. However, the clinical profile of patients and significance of indeterminate rifampin resistance (RR) results remain unclear.

**Table 1.**
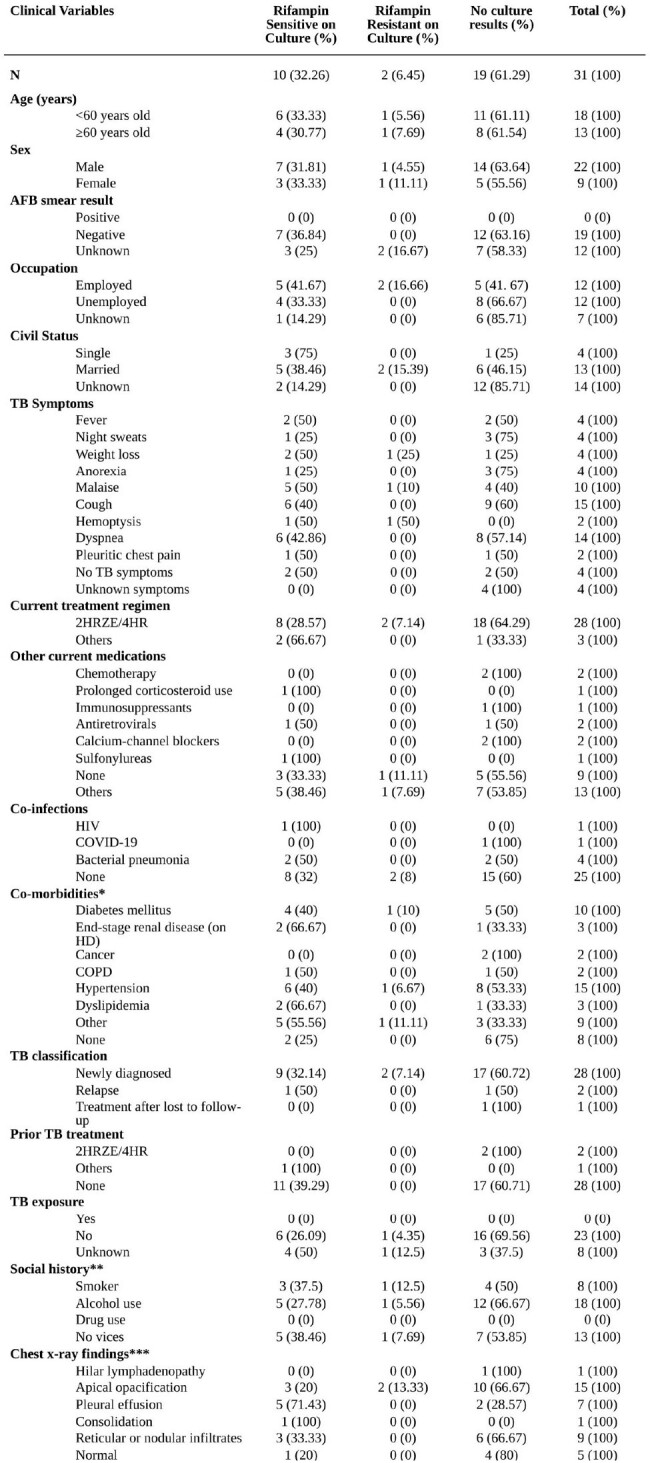
Clinical profile of Xpert RIF-Indeterminate patients

**Methods:**

Hospital and TB clinic databases from January 1, 2016–April 30, 2023 were reviewed to identify patients with both Xpert MTB/RIF positive TB and indeterminate RR results. The electronic health record and paper charts of cases with indeterminate RR were then retrieved for detailed review. Relevant demographic characteristics and laboratory results were collected for analysis. Descriptive statistics was performed using SPSS.

**Table 2.**
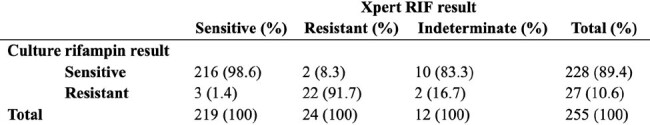
Concordance of Xpert and culture rifampin resistance results

**Results:**

Of 4339 records, only 31 cases (0.7%) fulfilled the criteria for study inclusion. The median age was 46 (range 22–97) years. Twenty-two patients were male (70.97%). The most common symptoms were cough (n=15, 48.39%), dyspnea (n=14, 45.16%), and malaise (n=10, 32.26%). Notably, only four had fever (12.90%). Four were asymptomatic. Majority were newly diagnosed TB (28/31, 90.32%) and had comorbidities (23/31, 74.2%), most commonly hypertension (n=15, 48.39%), diabetes mellitus (n=10, 32.26%), end-stage renal disease (n=3, 9.68%), and dyslipidemia (n=3, 9.68%). Eighteen patients reported alcohol use (58.06%). The most frequent chest x-ray findings were apical opacification (n=15, 48.39%), reticular or nodular infiltrates (n=9, 29.03%), and pleural effusion (n=7, 22.58%) (Table 1). Out of 31 patients, ten had rifampin-susceptible cultures (32.3%), two were rifampin resistant (6.4%), and the rest had unknown results (61.3%) (Table 2). Majority of patients were treated with standard 2HRZE/4HR regimen (28/31, 90.32%) and were alive (26/31, 83.87%) on the last follow up.

**Conclusion:**

Majority of patients with Xpert MTB/RIF indeterminate RR results had clinical symptoms and radiographic findings consistent with active TB. Patients with indeterminate RR results can have either susceptible or drug-resistant M. tuberculosis on culture. Indeterminate Xpert MTB/RIF RR results should be interpreted with caution, and final results of TB culture should be followed up.

**Disclosures:**

All Authors: No reported disclosures

